# Mutational signatures reveal mutual exclusivity of homologous recombination and mismatch repair deficiencies in colorectal and stomach tumors

**DOI:** 10.1038/s41597-023-02331-8

**Published:** 2023-07-01

**Authors:** Amir Farmanbar, Robert Kneller, Sanaz Firouzi

**Affiliations:** 1 Department of Computational Biology and Medical Sciences, Graduate School of Frontier Sciences, The University of Tokyo, Tokyo, Japan; 2 Research Center for Advanced Science and Technology, University of Tokyo, Minato-ku, Tokyo, 153-8904 Japan

**Keywords:** Cancer genomics, High-throughput screening

## Abstract

Decomposing somatic mutation spectra into mutational signatures and their corresponding etiologies provides a powerful approach for investigating the mechanism of DNA damage and repair. Assessing microsatellite (in)stability (MSI/MSS) status and interpreting their clinical relevance in different malignancies offers significant diagnostic and prognostic value. However, little is known about microsatellite (in)stability and its interactions with other DNA repair mechanisms such as homologous recombination (HR) in different cancer types. Based on whole-genome/exome mutational signature analysis, we showed HR deficiency (HRd) and mismatch repair deficiency (MMRd) occur in a significantly mutually exclusive manner in stomach and colorectal adenocarcinomas. ID11 signature with currently unknown etiology was prevalent in MSS tumors, co-occurred with HRd and was mutually exclusive with MMRd. Apolipoprotein B mRNA editing enzyme, Catalytic polypeptide-like (APOBEC) signature co-occurred with HRd and was mutually exclusive with MMRd in stomach tumors. The HRd signature in MSS tumors and the MMRd signature in MSI tumors were the first or second dominant signatures wherever detected. HRd may drive a distinct subgroup of MSS tumors and lead to poor clinical outcome. These analyses offer insight into mutational signatures in MSI and MMS tumors and reveal opportunities for improved clinical diagnosis and personalized treatment of MSS tumors.

## Introduction

Perturbation of cellular DNA damage response and repair systems leads to a high frequency of mutations^1,2^ and predisposes to development of cancer^3^. Emerging evidence indicates that inhibition of DNA repair pathways is a therapeutic option for targeted treatment of DNA repair-impaired cancers. Defects in one DNA repair pathway can be compensated by other pathways, suggesting that simultaneous defects in compensating pathways may result in synthetic lethality. Therefore, defects that occur in mutually exclusive patterns can be identified and employed for treatment of DNA repair-defective tumors^4,5^.

Homologous recombination (HR) is a multistep DNA repair process that is essential to the repair of DNA double-stranded breaks^6^. HR deficiency (HRd) is prevalent among various tumor types, especially in those of breast, ovaries, and pancreas, which as such are known as HRd-cancers^7–9^. HRd is a therapeutically actionable marker and a predictor of response to immunotherapy, chemotherapy and poly (ADP-ribose) polymerase inhibitor (PARPi) therapy^10^. Therefore, adequate assessment of HRd can improve outcome of such therapies. HRd is broadly defined from harboring deleterious alterations of HR pathway related genes to complex genomic scars^11^. Germline testing of BRCA1/2 bi-allelic inactivation is the current HRd assessment approach in clinic^12^. However, mechanisms of HRd extends beyond functional loss of BRCA1/2 indicating the need for more comprehensive evaluation approaches. To this end, HRD score was developed based on three independent factors: loss of heterozygosity (LOH)^13^, telomeric allelic imbalance (TAI)^14^, and large-scale state transitions (LST)^15^ to better indicate the extent of underlying genomic instability due to HR^16^. Discordant results of different HRd measuring approaches make patient stratification and therapeutic selection challenging^11^, which underscore the requirement for integrating efficient alternative measurement approaches.

The DNA mismatch repair (MMR) system plays an important role in maintaining genomic stability. The MMR system is primarily responsible for base-base mismatches and insertion/deletion mispaired nucleotides. Impairment of MMR limits correction of spontaneous mutations in microsatellites –i.e. DNA elements containing short repeating motifs– resulting in microsatellite instability (MSI) hypermutable tumor phenotype^17^. Accordingly, MSI-affected tumors may arise from a genetically or epigenetically perturbed MMR system. MSI-affected tumors, also known as MMR deficient (MMRd)-tumors, have been reported in diverse malignancies including colorectal, gastric and endometrial cancer etc., with varying MSI-positivity rates; colorectal and gastric cancers are among the most affected^18–22^.

Generally based on MSI status, colorectal and gastric cancers can be classified into two categories: (i) The microsatellite instability-high (MSI-H) group, which is caused by defects in the MMR system and accounts for ~15% of colorectal and ~10% of gastric tumors^18,23^. MSI-H tumors have a slightly better prognosis and do benefit from immune checkpoint blockade (ICB) therapy. (ii) The microsatellite stable (MSS) group, which exhibits chromosomal instability and accounts for the remaining ~85% and ~90% of colorectal and gastric tumors, respectively. MSS tumors are defined based on absence of MSI markers, are rarely sensitive to ICB therapy, and have limited treatment options^24–26^. Therefore, discovering the characteristics of MSS tumors and predicting their sensitivity to therapeutic agents is a need in the clinic today. Frequent silencing of HR and MMR pathways has been reported in gastrointestinal malignancies. In particular, colorectal cancer is known to have high mutational burden as well as high frequency of mutations in DNA damage and response pathway genes^16^. The presence and prevalence of HRd as well as its prognostic roles relative to levels of mismatch repair deficiency in colorectal and gastric tumors remains to be studied.

The total number of mutations presents in a tumor specimen is known as the tumor mutational burden (TMB) and has emerged as a novel therapeutic biomarker^27,28^. Factors contributing to high number of mutations, besides microsatellite instability, are not well-studied, and since TMB only represents the accumulation of somatic mutations, it does not provide evidence for underlying mechanisms. To fill this gap, mutational signatures that reflect both the patterns of mutations and their causative etiology can potentially elucidate relevant biological and mechanistic insights.

Diversity of somatic mutations can be decomposed into individual mutational signatures, describing patterns of mutagenesis that arise because of DNA damage and defective DNA repair processes. By considering the entire coding and non-coding catalogs, mutational signatures have become a powerful tool for identifying processes that generate somatic mutations during tumorigenesis in different cancer types^29–31^. In this study, we comprehensively investigated mutations occurring in colorectal and gastric tumors. This revealed distinct mutational signature profiles associated with clinical outcome in these two common MMRd-cancer types.

## Methods

### Mutation data source and cleaning

We accessed, cleaned, and reformatted Simple Somatic Mutation (SSM) files for whole-exome sequencing (WES) and whole-genome sequencing (WGS) of STAD and COAD samples from the data portal of the International Cancer Genome Consortium (ICGC, https://dcc.icgc.org/projects). In brief, Mutations including Single-Base Substitutions (SBSs), Double-Base Substitutions (DBSs) and small Insertions/Deletions (IDs) were extracted from SSM files, converted to mutation matrices and utilized for subsequent analyses. The raw data is publicly accessible via ICGC portal using each unique Sample ID. Information regarding the entire sample set and processed data are provided in Supplementary Table S1(A–X).

The MSI-H/MSS status for WES of stomach adenocarcinoma (STAD) tumors were retrieved and quality-checked for MSI-H (n = 25) and MSS (n = 73); and colorectal adenocarcinoma (COAD) tumors for MSI-H (n = 42) and MSS (n = 164) from two independent sources^32,33^. We used the samples that had consistent and reliable MSI-H and MSS status available. The SSM files for the WGS data of 75 STAD and 90 COAD tumors from the ICGC portal were obtained similarly.

HRd, LOH, TAI and LST scores were retrieved from^16^ (https://gdc.cancer.gov/about-data/publications/PanCan-DDR-2018).

### Mutational signatures analysis

Using GRCh37 and Catalogue of Somatic Mutations in Cancer (COSMIC, V3.1) (http://cancer.sanger.ac.uk/cosmic/signatures), a non-Negative Matrix Factorization (NMF)-based *de novo* mutational signatures analysis^34,35^ (https://github.com/alexandrovlab) were run over WES and WGS of tumor samples.

In addition, the computational tool SigMA^36^ using hg19 was applied for confirmatory mutational signature analysis. SigMA uses a multivariate approach to accurately detect the mutational signature associated with HR deficiency (SBS3) from WGS, WES and targeted gene panels, even from low mutation counts.

The first and second dominant signatures were extracted and visualized based on their contributions over WES /WGS data of STAD and COAD tumors. R packages of “car”, “ComplexHeatmap”, “vioplot”,“circlize”, and “ggplot”^37–39^ were used for visualization. Consensus molecular subtype (CMS) for COAD tumors were annotated as described in^40,41^.

### Survival analysis

Progression-free survival (PFS) and overall survival (OS) data were downloaded from cbioportal (https://www.cbioportal.org/). R packages “Survminer” and “survival”^42^ were used for survival analysis.

### Statistical analyses

P values were calculated as appropriate for continuous or categorical variables using Mann Whitney U test, Hypergeometric test or Fisher’s exact test were used. All analyses were conducted in the R statistical environment (R version 4.0.0 http://www.r-project.org/). All reported *P* values were two tailed; ≤0.05 was considered significant.

## Results

We performed mutational signature analyses using two independent and complementary methods. First, we used a NMF-based *de novo* mutational signature detection approach, decomposed by the COSMIC, V3.1, and analyzed SBSs, IDs, and DBSs detected by WES of MSI-H (n = 25) and MSS (n = 73) STAD, and MSI-H (n = 42) and MSS (n = 164) COAD tumors as well as WGS of STAD (n = 75) and COAD (n = 90) samples.

### Mutational signatures of MSI-H exomes reveal MMRd signatures

**Fig. 1:**
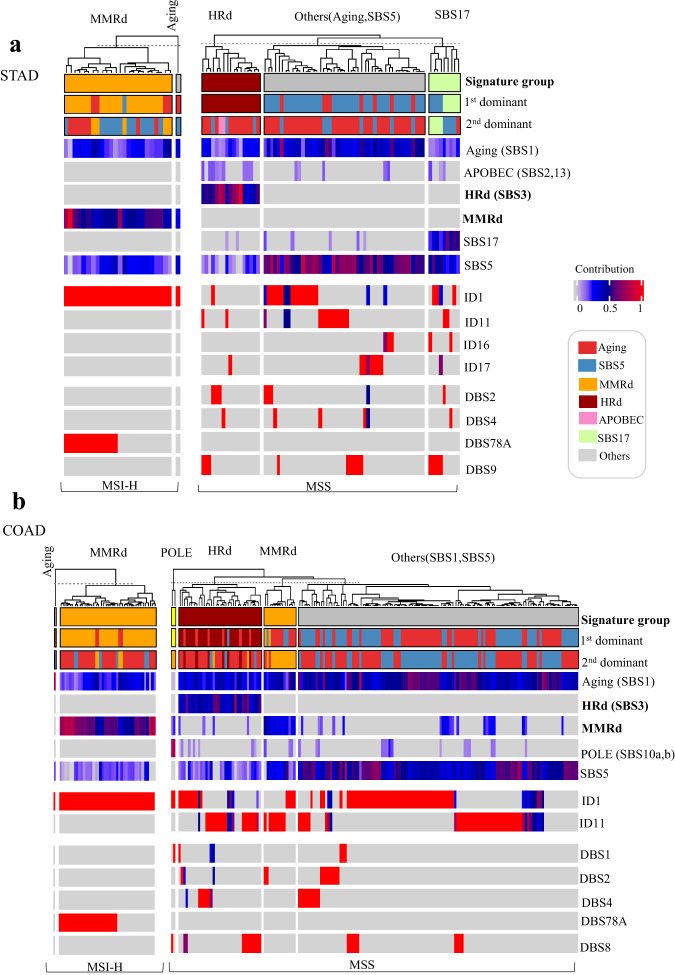
Mutational signature analysis of WES data from MSI-H and MSS STAD and COAD tumors. (**a,b**) NMF-based *de novo* mutational signatures of STAD (**a**) and COAD (**b**) tumors visualized by a heatmap divided based on distinct signature status. The first and second dominant signatures are annotated at the top. STAD and COAD tumors’ MSI-H and MSS status are annotated at the bottom. Hierarchical clustering was performed based on the relative contribution of signatures in each tumor. Color codes represent each mutational signature shown. The color scale shows the contribution values of each mutational signature. For mutational signatures with known etiology, both signature and etiology are indicated.

**Fig. 2:**
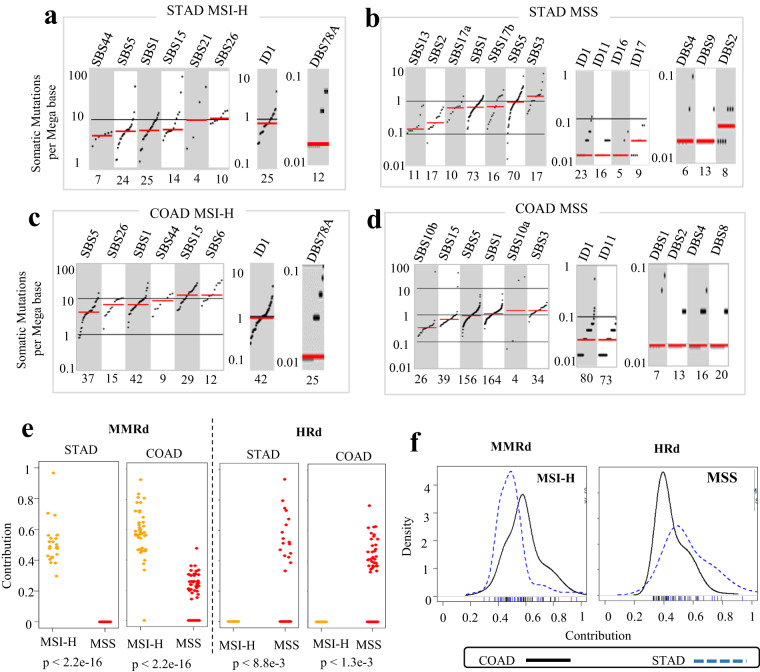
TMB and contribution of mutational signatures in WES data from MSI-H and MSS STAD and COAD tumors. (**a**–**d**) TMB of SBS and ID and DBS signatures for MSI-H and MSS STAD and COAD tumors, respectively. TMB is measured in somatic mutations per Megabase (Mb). In the TMB plots, columns represent the detected mutational signatures and are ordered by mean somatic mutations per Mb from the lowest frequency, left, to the highest frequency, right. Numbers at the bottom of the TMB plots represent the numbers of tumors harboring each mutational signature. Only samples with counts more than zero are shown. (**e**) Relative contribution of the MMRd and HRd signatures identified among MSI-H and MSS tumors in STAD and COAD. Each point represents the value of contribution detected in individual tumor specimens. *P* values were calculated by two-tailed Mann Whitney U test. (**f**) Distribution of relative contribution for MMRd signature in MSI-H tumors (left side) and HRd signature in MSS tumors (right side) shown in density plots. The X axis represents contribution. Ticks for each contribution value are presented under the distribution curves. The Y axis is a kernel density estimate to show the probability density.

We detected MMRd SBS signatures in all MSI-H STAD tumors, including SBS15, SBS21, SBS26 and SBS44. The only other signatures in these tumors were SBS1 and SBS5 (Fig. 1a). Similarly, we detected MMRd signatures, including SBS6, SBS15, SBS26 and SBS44, in all but one of the MSI-H COAD tumors, in addition to SBS1 and SBS5 signatures (Fig. 1b). SBS26 in MSI-H STAD and SBS6 MSI-H COAD tumors, had the highest TMB with a median of >10 somatic mutations per Megabase (Fig. 2). MMRd SBS signature had significantly higher contribution in MSI-H compared to MSS tumors in both STAD and COAD (Fig. 2e, left). In contrast, the HRd signature(SBS3) had a significantly higher contribution in MSS tumors compared to MSI-H tumors in both STAD and COAD (Fig. 2e, right). The distribution shapes of MMRd signatures in MSI-H STAD and MSI-H COAD tumors are compared as density plots in (Fig. 2f). The average contribution value of MMRd signature was 0.51 (range: 0.30–0.97) in MSI-H STAD compared to 0.59 (range: 0.33–0.93) in MSI-H COAD tumors.

We detected the ID1 signature in all MSI-H STAD and COAD tumors with a median TMB of ~1 somatic mutation per Megabase (range: 0.1–10), one-tenth of the TMB measured for the SBS signatures (range: 0.1–100) (Fig. 2). Replication slippage has been suggested as the etiology of ID1^31^.

Both MSI-H STAD and MSI-H COAD tumors showed DBS78A. In MSI-H STAD tumors, DBS78A had cosine similarity of 0.57 with COSMIC’s DBS4 and DBS11. In MSI-H COAD tumors, DBS78A had cosine similarity of 0.75 with DBS4 and DBS10. According to^31^, etiology of DBS10 and DBS11 signatures is associated with MMRd and, respectively, Apolipoprotein B mRNA editing enzyme, Catalytic polypeptide-like (APOBEC); while the etiology of DBS4 is unknown (Figs. 1, 2). DBS signatures are difficult to interpret because they have relatively low mutation counts, and based on our results, their pattern might be shaped by a combination of different etiologies. Supplementary Table S1.

### Mutational signatures of MSS exomes reveals distinct group of patients with HRd signature

In 23% of MSS STAD and 21% of MSS COAD tumors, we detected the SBS3 signature, which is caused by HRd^8,31^ (Fig. 1a,b). In both MSS STAD and MSS COAD tumors, SBS3 had the highest TMB with medians of >1 somatic mutation per Megabase (Fig. 2b,d). The average contribution value of HRd signature was 0.57 (range: 0.33–0.94) in MSS STAD compared to 0.46 (range: 0.33–0.77) in MSS COAD tumors (Fig. 2e,f).

Mutational signatures in the MSS STAD tumors without SBS3 consisted of SBS17 whose etiology is unknown (Fig. 1a). In the MSS COAD tumors without SBS3, a subset of samples was dominated by the SBS10a, b (POLE) mutation signatures. In 24% of MSS COAD tumors, we detected the SBS15 signature, which is associated with the MMRd etiology (Fig. 1b).

MSS STAD tumors had ID1 and ID11 as well as ID16 and ID17 while MSS COAD tumors only had ID1 and ID11. Although the etiology of the ID1 signature is known as replication slippage, etiologies of ID11, ID16, and ID17 remain unknown (Fig. 1a,b). Recently, defects in topoisomerase without defective mismatch repair is proposed as the etiology of ID17^43^. Our data also supports that ID17 is only detected in a subset of MSS but not in MSI tumors.

MSS STAD tumors showed DBS2, DBS4, and DBS9, whereas MSS COAD tumors showed DBS1, DBS2, DBS4 and DBS8. The etiologies of these signatures remain unknown (Fig. 1a,b). The TMB range was 0.01–10 and 0.01–100 per SBS signature in MSS STAD and MSS COAD tumors, respectively. In contrast, the TMB was 0.01–1 per ID signature. The TMB range was 0.01–1 per DBS signature for both MSS STAD and MSS COAD tumors (Fig. 2b,d) Supplementary Table S1.

### Dominant signatures distinguish MSS and MSI-H tumors

**Fig. 3:**
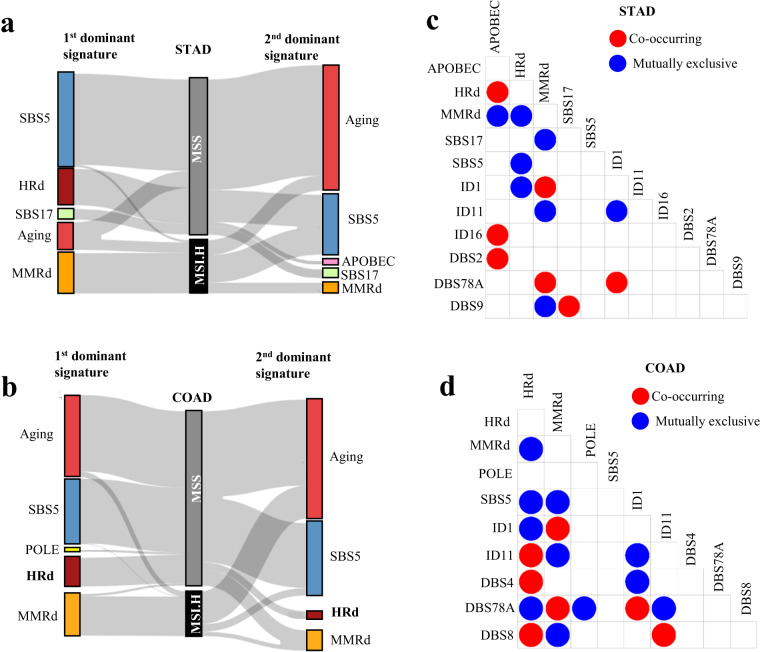
Dominant signatures and interaction analysis of WES data from MSI-H and MSS STAD and COAD tumors (**a,b**) The first and second dominant SBS mutational signatures in MSI-H and MSS STAD and COAD tumors. Dominant signatures were based on the contribution value of detected mutational signatures. (**c,d**) Pair-wise statistical interaction of signatures measured by hypergeometric test: red represents co-occurrence and blue represents mutual exclusivity.

All cases of MSI-H STAD tumors but one had MMRd as their first or second dominant signature. MMRd SBS signatures were the first dominant signature in 76%, and the second dominant signature in 20% of MSI-H STAD tumors. In 24% of MSI-H STAD tumors in which MMRd signatures were not the first, SBS1 and SBS5 were the first and/or second dominant signatures (Fig. 3a).

In 23% of MSS STAD tumors, HRd was the first dominant signature; the remaining tumors had SBS1, SBS5, and SBS17 as their first dominant signature. SBS1, SBS5, SBS2 and 13 (APOBEC), and SBS17 signatures were second dominant signatures of MSS STAD tumors. Of note, wherever present, HRd was the first dominant signature in all cases of MSS STAD tumors (Fig. 3b).

MSI-H STAD tumors had only ID1 as the dominant ID signature. MSS STAD tumors had ID1, ID11, ID16, and ID17 as the first dominant ID signatures, and ID1 and ID11 as second dominant ID signatures (Supplementary Fig. S1a). Considering the number of SBS and ID signatures harbored, MSS STAD tumors were more heterogeneous compared to MSI-H STAD tumors (Fig. 3a,b).

MSI-H STAD tumors had only DBS78A (Cosine similarity of 0.57 with DBS4 and DBS11) as their DBS dominant signature. MSS STAD tumors had DBS2, DBS4, and DBS9 as their first dominant and DBS4 as their second dominant DBS signatures (Supplementary Fig. S1b).

Consistent with the pattern observed in STAD tumors, MMRd signatures were the first dominant SBS signature in 88%, and the second dominant signature in the remaining MSI-H COAD tumors. In 12% of MSI-H COAD tumors in which MMRd signatures were not the first dominant, SBS1 (aging) was the first dominant SBS signature. The second dominant signatures of COAD tumors were SBS1, SBS5, and MMRd. In all cases of MSI-H COAD tumors, MMRd was the first or second dominant SBS signature, highlighting the ability of NMF-based *de novo* mutation signature analysis to detect the MMRd status of MSI-H tumors (Fig. 3a,b).

HRd (SBS3) was the first dominant signature in 16.5% of MSS COAD tumors; SBS1, SBS5, and SBS10a, b (POLE) were the first dominant in the remaining. HRd was the second dominant SBS signatures in 4.3% of MSS COAD tumors with SBS1, SBS5 and MMRd in the remaining. HRd, wherever presented, had the highest TMB and was the first or second dominant signature among MSS COAD tumors (Fig. 3a,b).

As noted earlier, MSI-H COAD tumors had only ID1 as the dominant ID signature. MSS COAD tumors had ID1 and ID11 as the first and second dominant signature (Supplementary Fig. S1c). Considering the number of SBS and ID signatures harbored, MSS COAD tumors were more heterogeneous than MSI-H COAD tumors, similar to MSS STAD tumors (Fig. 3).

MSI-H COAD tumors had only DBS78A (cosine similarity of 0.75 with DBS4 and DBS10) as their dominant signature. However, MSS COAD tumors had DBS1, DBS2, DBS4, DBS8 as the first dominant and DBS1, DBS2, DBS4 as the second dominant signatures (Supplementary Fig. S1d).

Association analysis between CMS of COAD tumors and mutation signatures showed CMS1 and CMS2 were significantly enriched in MSI-H.MMRd and MSS-HRd tumors respectively (Fisher’s exact test, p = < 0.01e-3) (Supplementary Fig. S1e).

### Mutational signatures of STAD and COAD WES tumors reveal mutual exclusivity of MMRd and HRd signatures

In both STAD and COAD WES tumors, MMRd and HRd signatures occurred in a mutually exclusive manner. In STAD WES tumors, like HRd, the APOBEC signatures were mutually exclusive with MMRd. In STAD tumors, DBS9 was mutually exclusive with MMRd and co-occurred with SBS17. In addition, SBS17 was mutually exclusive with MMRd signatures in STAD WES tumors. Notably, ID11 with unknown etiology was mutually exclusive with MMRd in both tumors while co-occurred with the HRd(SBS3) in COAD. Similarly, DBS4 and 8 co-occurred with the HRd(SBS3) in COAD (Fig. 3c,d).

### MSS tumors with HRd had the poorest patient outcome and showed higher HRd scores

**Fig. 4:**
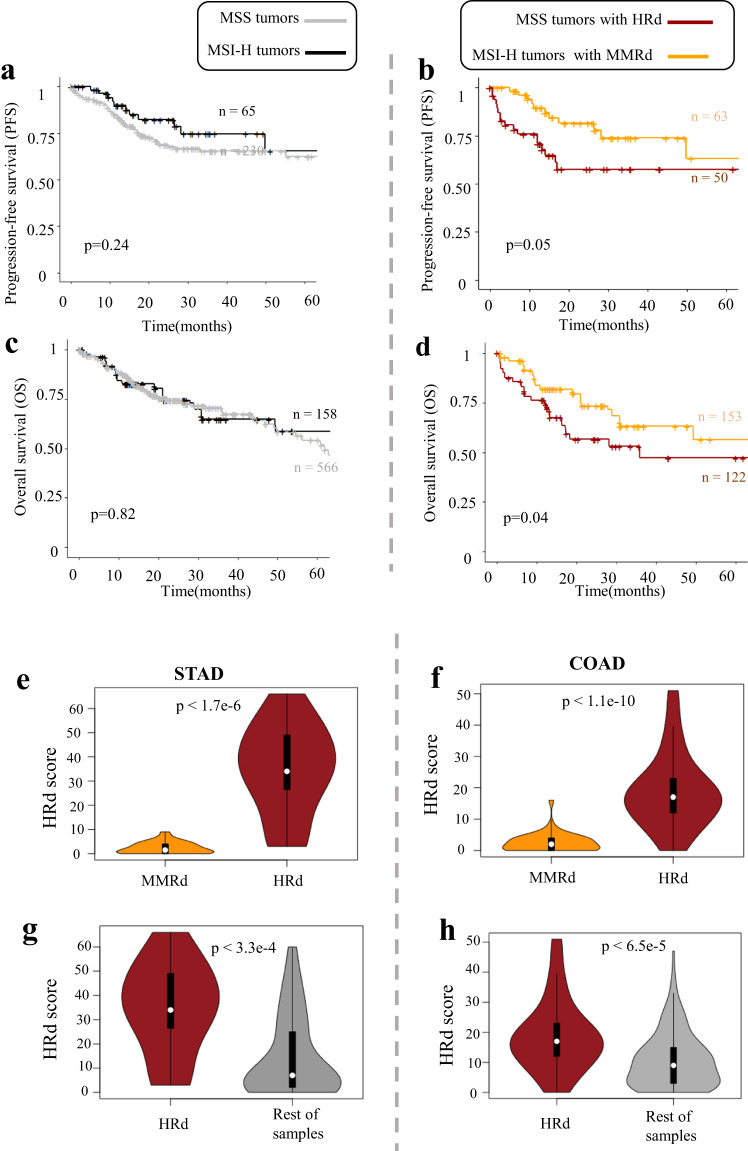
Comparing MMRd and HRd signatures and survival of MSI-H and MSS tumors. (**a**) Kaplan-Meyer curves representing PFS of patients (aggregated COAD and STAD tumors), comparing MSI-H *vs*. MSS tumors. (**b**) Patients’ PFS for the same tumors in a, stratified by harboring MMRd and HRd signatures (MSI-H tumors with MMRd, vs. MSS tumors with HRd). (**c**) Kaplan-Meyer curves representing OS of patients (aggregated COAD and STAD tumors), comparing MSI-H *vs*. MSS tumors. (**d**) Patients’ OS for the same tumors in a, stratified by harboring MMRd and HRd signatures (MSI-H tumors with MMRd, vs. MSS tumors with HRd). P values represent the significance determined from log-rank. (**e,f**) Comparing HRd score of tumors with MMRd and HRd signatures in STAD (**e**) and COAD (**f**). (**g,h**) Comparing HRd score of tumors with HRd signatures and rest of cases in STAD (**g**) and COAD (**h**). P values represent the significance determined from Mann Whitney U test.

Consistent with previous reports that MSI-H status is associated with slightly improved patient outcome^44^, we did not observe significant differences in progression-free survival (PFS) of MSI-H and MSS tumors (Fig. 4a). Median PFS times were 19 months for MSI-H and 18 months for MSS tumors. PFS rates at 20 months were 43% for MSI and 40% for MSS tumors (Fig. 4b). However, we did find survival differences between MSI/MSS status when differentiating according to MMRd and HRd signatures. Figure 4b indicates significantly higher likelihood of PFS for MSI-H tumors with MMRd signatures compared to MSS tumors with the HRd signature, at least within the first five years after tumor excision. PFS at 20 months were 43% for MSI-H tumors with MMRd compared to only 28% for MSS tumors with HRd. Median PFS times for MSI-H tumors with MMRd, MSS tumors with HRd and other tumors were 19,13,18 months respectively. (Fig. 4b). OS data were consistent with PFS (Fig. 4c,d) (Supplementary Fig. S2). Because there were few survivors at 60 months, the uncertainly ranges prevent comparison of data beyond 5 years.

To further confirm the HRd status of identified MSS tumors with HRd signature, we compared HRd, LOH, TAI, LST scores which are the measures of genomic instability. In both STAD and COAD, MSS tumors with HRd had significantly higher HRd, LOH, TAI, LST scores compared to MMRd tumors and the rest of tumors (Fig. 4e–h) (Supplementary Fig. S2c–h).

### Mutational signature multivariate analysis of MSS and MSI-H tumors portrays HRd subgroup in MSS tumors

As a second approach, and to further confirm the results of NMF-based *de novo* mutation signatures, we used a complimentary, multivariate method for extracting SBS mutational signature according to COSMIC V2.0.

Consistent with *de novo* mutation signatures, this approach confirmed the presence of a subgroup of MSS tumors that exhibit the HRd signature and verified the absence of the HRd signature in MSI-H tumors for both STAD and COAD. We also further confirmed that HRd was highly prevalent and was detected as dominant in 27% of STAD MSS, and in 14% of COAD MSS tumors (Supplementary Figs. S3, S4). These results suggested that MMRd and HRd occur in a mutually exclusive manner with no overlap between groups. We also detected SBS18 as a Reactive Oxygen Species (ROS) etiology signature in MSS tumors of both STAD and COAD. It was not detected by *de novo* mutational signature analysis. Interestingly, we observed the POLH –i.e. SBS9 (AID)– signature in a few MSS STAD tumors. MSI-H tumors showed only SBS1, SBS5, and MMRd signatures while MSS tumors showed a combination of SBS1, SBS5, HRd, and ROS signatures. In both STAD and COAD, heterogeneity of signatures in MSS tumors was higher than in MSI-H tumors. Multivariate analysis also confirmed the pattern of co-occurrence and exclusiveness among HRd, APOBEC and MMRd (Supplementary Figs. S3b, S4b).

### Analysis of WGS data reveals presence of distinct groups of MMRd and HRd

**Fig. 5:**
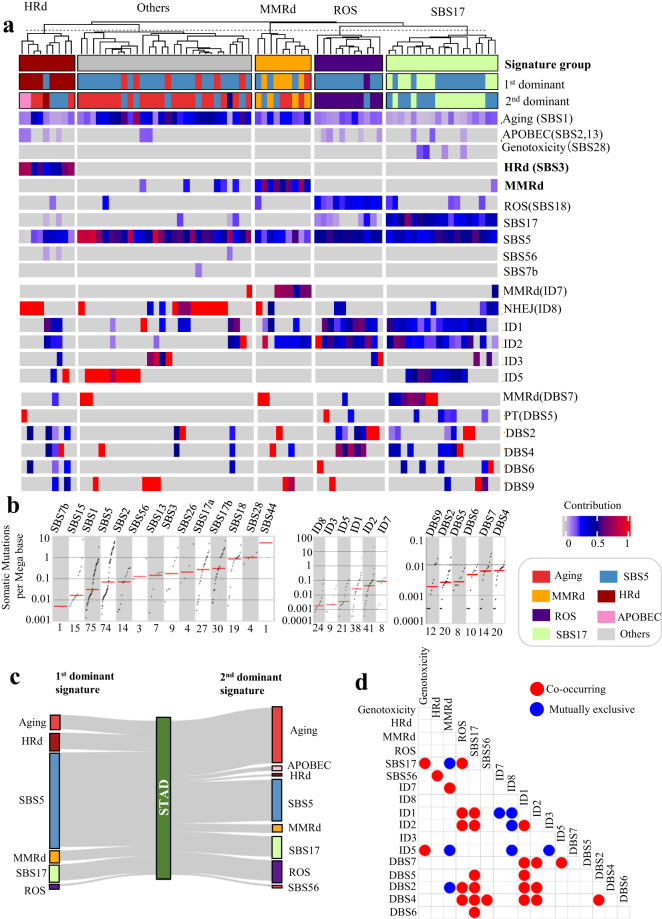
NMF-based mutational signature analysis of WGS data from STAD tumors. (**a**) NMF-based *de novo* mutational signatures of STAD WGS tumors visualized by a heatmap divided based on distinct signature status. The first and second dominant signatures are annotated at the top. Hierarchical clustering was performed based on the relative contribution of signatures in each tumor. Color codes represent each mutational signature shown. The color scale shows the contribution values of each mutational signature. For mutational signatures with known etiology, both signature and etiology are indicated. (**b**), (**c**) TMB of SBS and ID and DBS signatures for STAD tumors, respectively. TMB is measured in somatic mutations per Megabase (Mb). In the TMB plots, columns represent the detected mutational signatures and are ordered by mean somatic mutations per Mb from the lowest frequency, left, to the highest frequency, right. Numbers at the bottom of the TMB plots represent the numbers of tumors harboring each mutational signature. Only samples with counts more than zero are shown. (**d**) The first and second dominant SBS mutational signatures in STAD tumors. Dominant signatures were based on the contribution value of detected mutational signatures. (**e**) Pair-wise statistical interaction of signatures measured by hypergeometric test: red represents co-occurrence and blue represents mutual exclusivity.

Next, we applied the mutational signature analyses described above to the WGS data from STAD and COAD tumors. Twenty percent of STAD tumors had MMRd SBS signature including SBS15, SBS26, and SBS44. A 12% (9 out of 75) subset of STAD tumors had SBS3 as the HRd signature and 24% had only SBS1 and SBS5 signatures. The remaining 44%, in addition to SBS1 and SBS5, mainly exhibited a combination of ROS, SBS28, and SBS2,13 (APOBEC). SBS44 followed by SBS28, SBS18 (ROS), and SBS17 showed the highest TMB (Fig. 5). Colibactin genotoxin has been suggested as a possible etiology of SBS28^45^.

**Fig. 6:**
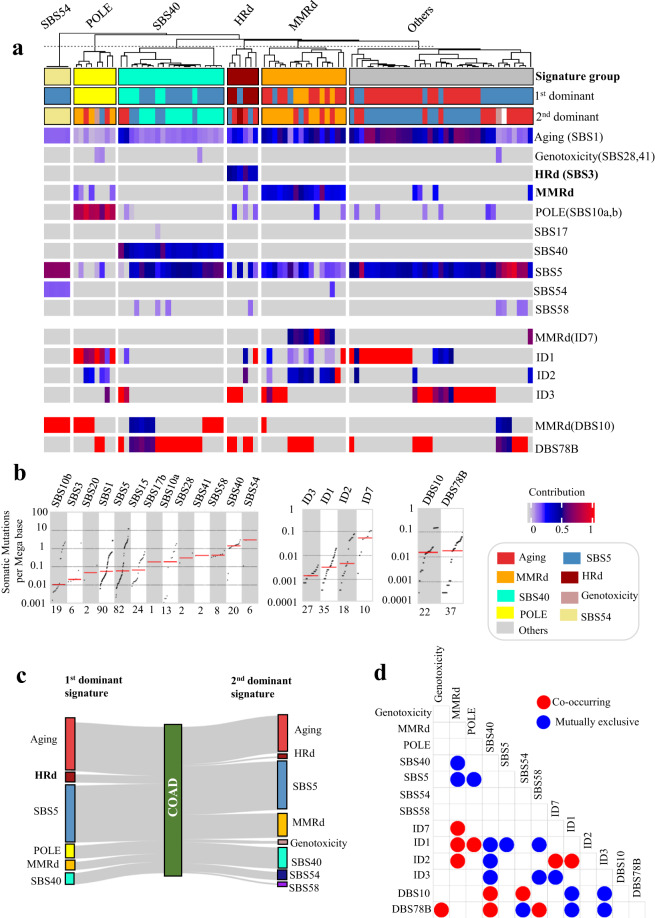
NMF-based mutational signature analysis of WGS data from COAD tumors. (**a**) NMF-based *de novo* mutational signatures of COAD WGS tumors visualized by a heatmap divided based on distinct signature status. The first and second dominant signatures are annotated at the top. Hierarchical clustering was performed based on the relative contribution of signatures in each tumor. Color codes represent each mutational signature shown. The color scale shows the contribution values of each mutational signature. For mutational signatures with known etiology, both signature and etiology are indicated. (**b**), (**c**) TMB of SBS and ID and DBS signatures for COAD tumors, respectively. TMB is measured in somatic mutations per Megabase (Mb). In the TMB plots, columns represent the detected mutational signatures and are ordered by mean somatic mutations per Mb from the lowest frequency, left, to the highest frequency, right. Numbers at the bottom of the TMB plots represent the numbers of tumors harboring each mutational signature. Only samples with counts more than zero are shown. (**d**) The first and second dominant SBS mutational signatures in COAD tumors. Dominant signatures were based on the contribution value of detected mutational signatures. (**e**) Pair-wise statistical interaction of signatures measured by hypergeometric test: red represents co-occurrence and blue represents mutual exclusivity.

These analyses also showed that 27% of COAD tumors had MMRd signatures, including SBS15 and SBS20. A 7% (6 out of 90) subset of COAD tumors had the HRd signature, 20% showed the SBS10a, b (POLE) signature, while the rest mainly showed a combination of SBS1, SBS5, SBS40. SBS54 followed by SBS 40 and SBS58 had the highest TMB (Fig. 6).

Both STAD and COAD tumors had a group of cases (11%) with the ID7 (MMRd) signature, which also had the highest ID TMB (Fig. 6, Fig. 6). ID1, ID2, ID3 were present in both COAD and STAD. We also detected ID5 with unknown etiology in STAD tumors. Interestingly we detected ID8, a marker of non-homologous end joining (NHEJ), in these tumors further highlighting the role of HRd in STAD (Figs. 5, 6).

STAD tumors showed combination of DBS2, DBS4, DBS6, DBS9, DBS7 (MMR), and DBS5 associated with prior platinum therapy (PT). DBS4 had the highest TMB. COAD tumors showed DBS78B with cosine similarity <0.5 with COSMIC’s DBS7 and DBS11. The proposed etiology of DBS7 and DBS10 are MMRd (Figs. 5, 6).

Dominant signature analysis of WGS results also confirmed the presence of the HRd subgroup with patterns that were consistent with WES data. Similar to WES, SBS17 was mutually exclusive with MMRd signatures in STAD WGS tumors. In addition, DBS10 and SBS54 co-occurred in COAD WGS tumors, suggesting possible MMRd etiology (Figs. 5c,d, 6c,d, Supplementary Fig. S5).

### Signature multivariate analysis of WGS data confirms MMRd and HRd groups

We also applied a complimentary multivariate method to extract SBS signatures, as described above. We detected and distinguished tumors with MMRd and HRd signatures in an unsupervised manner and without the knowledge of the samples’ MSI or MSS molecular subtypes. In both STAD and COAD, MMRd and HRd signatures were mutually exclusive. MMRd signatures were dominant among 8% of STAD and 26% of COAD tumors, while HRd signatures was dominant among 24% of STAD and 7% of COAD tumors (Supplementary Figs. S6, S7).

Consistent with *de novo* mutation signatures, in both STAD and COAD, mutational signature heterogeneity of signatures in the subset of tumors with MMRd was lower than the rest of tumors. Moreover, we detected the SBS18(ROS) signature using the multivariate method in both STAD and COAD; and observed POLH, i.e. SBS9(AID) signature in a subset of STAD tumors Notably, multivariate analysis of WGS tumors also confirmed the pattern of co-occurrence and exclusiveness among HRd, APOBEC and MMRd (Supplementary Figs. S6, S7).

## Discussion

We uncovered possible diagnostic and prognostic roles of mutational signatures as a biomarker for stratification of HRd in MSS tumors. Our findings provide relevant biological insights regarding the relationship between the tumors’ MSI/MSS status and presence of HRd. We found that MSS tumors had a greater degree of heterogeneity in their mutational signatures compared to MSI-H tumors. We showed that MSI-H tumors harbored SBS signatures that faithfully reflected a strong fingerprint of MMRd, which dominated the mutational signature spectra, and suggested selective advantages of these signatures and their possible driver role in shaping tumors mutational landscape. Notably, our findings revealed mutual exclusivity of MMRd and HRd mutational signatures in colorectal and stomach cancers, which is consistent with previous report on gynecological malignancies^20^.

Although there are appropriate targeted therapies available for MSI-H tumors, treatment options for patients with MSS tumors, which constitute the majority of COAD and STAD cases, have been limited. This is largely due to the fact that MSS tumors are highly heterogeneous, and their genetic and molecular characteristics remain to be fully characterized. To prevent patients from receiving incorrect treatment or missing out on possible treatment opportunities, development of better biomarkers and therapies for MSS patients is necessary. HRd targeted therapy with PARPi is mostly used for treating breast and ovarian cancers^12^, and was recently approved by the US Food and Drug Administration for treatment of pancreatic cancers^46^. Our results strongly suggest that the presence of HRd signature can be used as actionable marker to identify a distinct subset of MSS STAD and COAD tumors which may benefit from PARPi therapy. Additionally, as several studies reported HRd patients with diverse cancer types are sensitive to platinum-based chemotherapies^47,48^, a distinct subset of COAD and STAD MSS tumors with HRd mutational signature may also benefit for this therapeutic regimen.

Previous studies reported that WES can detect clonal mutational signatures that are active in the majority of cancer cells, while WGS is required to extract subclonal mutational signatures^49,50^. Accordingly, we propose that ID11 and SBS6, which have relatively high TMB and are observed in WES data, are clonal. In contrast, ID7 and SBS54, which also have high TMB, co-occur with other MMRd signatures, and are commonly detected in WGS data, may be active subclonally. WES may not have the power and/or the scope to detect such signatures. Accordingly, it is reasonable to suggest that SBS18, which is an effective signature present in STAD tumors, occurs mainly across the whole genome. Collectively, these results suggest that both WGS and WES can generate reliable mutational signature profiles while WGS data are superior for detecting particular mutational signatures that may affect the non-coding more than the coding genome. Therefore, the appropriate input data for mutational signature analysis can be selected depending on the purpose of experiments.

Extraction of SBS3, 5, 8, and 40, which are known as flat signatures, by NMF-based *de novo* method is often mathematically challenging^31^. Our analyses using SigMA^36^, which is based on a multivariate analysis, showed consistent results for both methodologies and demonstrated that HRd and MMRd signatures were robustly detected by either approach. Consistency of HRd scores with the mutational signature results further confirms presence of a subset of MSS tumors with HRd signature. Unlike our study which considers the entire coding and non-coding mutations to stratify tumors using mutation signature profiles, a recent study employed mutation in limited numbers of preselected HR genes to define HRd status of tumors^51^. They reported HRd tumors to be more frequent among MSI than MSS tumors. Their data contradicts ours and several other comprehensive studies^16,20^. Moretto *et al*. using colorectal tumors without normal match samples could not differentiate biallelic/monoallelic and germline/somatic alterations which leaded to overestimation of HRd status and mis-annotation of a high percentage of MSI tumors as HRd. Several studies have demonstrated that not all HR gene mutations result in identical consequences and only biallelic inactivation of many HR genes are functional and reflect underlying DNA-repair deficiency^16,52,53^. Therefore, simple annotation of tumors based on presence or absence of mutations without differentiating biallelic/monoallelic and deleterious/passenger alterations leads to erroneous prediction of HRd status.

Notably, we detected SBS9 in STAD but not in COAD tumors. SBS9 is characterized by patterns of mutations that contribute to POLH i.e. AID. POLH encodes for a specialized error-prone polymerase which promotes somatic hypermutation in the variable regions of immunoglobulin genes. Dysregulation and mistargeting of POLH can compromise genome integrity^54^. POLH signature has been reported in different cancer types^55,56^, but has been mainly studied in hematological malignancies^57^. Helicobacter pylori infection has also been suggested to trigger aberrant expression of AID, which induces mutations that may lead to gastric carcinogenesis^58^. Clinical and biological roles of the POLH signature in STAD tumors, and whether there is an association between Helicobacter pylori infection and the POLH signature remains to be studies.

Several studies have demonstrated that SBS mutational signatures may serve as prognostic or predictive biomarkers across different cancer types^9,59,60^. Collectively, we propose that the understanding of the mechanistic basis of mutational signatures, as well as their etiology, improves cancer diagnosis and holds prognostic value. Effective determination of MSI/MSS as well as HRd status in different cancer types including gastric and colorectal tumors may improve selection of appropriate therapy with implications for specialized management of patients. We believe further studies are necessary to fully investigate the biological and clinical impact of MMRd and HRd in all malignancies.

Taken together, we presented complementary mutational signature analyses of stomach and colorectal adenocarcinomas using WES and WGS data. We showed that deficiency in HR and MMR result in mutually exclusive mutational signatures. Mutational signatures in MSI tumors were dominated by those caused by MMR deficiency; while mutational signatures in MSS tumors were diverse and identified a distinct group of HR-deficient tumors with the poorest patient outcome. Etiologies detected by our mutational signature analysis, including the prevalence of HR-deficiency, provide a means for selection of therapeutic targets and increasing positive outcomes for cancer patients.

## Supplementary information

Supplementary information

## Data Availability

All data generated or analyzed during this study are included in this published article and its supplementary information files. Supplementary table S1 is deposited on Figshare (10.6084/m9.figshare.22818080)^61^. All R packages and scripts used for the analyses are available publicly as described in the methods section. ICGC dataset is available at https://dcc.icgc.org/projects,

## Code availability

No custom code was used.
