# Supplementary material for: Mutational signatures reveal mutual exclusivity of homologous recombination and mismatch repair deficiencies in colorectal and stomach tumors
Source: Sci Data. 2023 Jul 1;10:423. doi: 10.1038/s41597-023-02331-8 (PMC10314920; doi:10.1038/s41597-023-02331-8)

SUPPLEMENTARY INFORMATION

Mutational signatures reveal mutual exclusivity of homologous recombination and mismatch repair deficiencies in colorectal and stomach tumors

Amir Farmanbar[1,2*], Robert Kneller[2], Sanaz Firouzi[1*#]

Supplementary Figure legends

Supplementary Fig. S1. Dominant signature analysis of WES data among MSI-H and MSS tumors. **(a)** The first and second dominant ID mutational signatures in MSI-H and MSS STAD tumors. **(b)** The first and second dominant DBS mutational signatures in MSI-H and MSS STAD tumors. **(c)** The first and second dominant ID mutational signatures in MSI-H and MSS COAD tumors. **(d)** The first and second dominant DBS mutational signatures in MSI-H and MSS COAD tumors**.** Dominant signatures were based on the contribution value of detected mutational signatures. For mutational signatures with known etiology, both signature and etiology are indicated. **(e)** COAD Consensus molecular subtype (CMS) of each major group of MSI-H.MMRd and MSS.HRd.

Supplementary Fig. S2. Comparing PFS, OS, TIA, LST and LOH of MSI-H tumors with MMRd, MSS tumors with HRd and other tumors. (a) Kaplan-Meyer curves representing PFS of patients (aggregated COAD and STAD tumors) **(b)** Kaplan-Meyer curves representing OS of patients (aggregated COAD and STAD tumors). P values represent the significance determined from log-rank. **(c,d)** TAI score of tumors with MMRd and HRd signatures in STAD(c) and COAD(d). **(e,f)** LST score of tumors with MMRd and HRd signatures in STAD(e) and COAD(f). **(g,h)** LOH score of tumors with MMRd and HRd signatures in STAD(g) and COAD(h). P values represent the significance determined from Mann Whitney U test.

Supplementary Fig. S3. Mutational signature analysis of WES data from MSI-H and MSS STAD tumors with multivariate approach. (a) Mutational signatures of STAD tumors visualized by a heatmap divided based on distinct signature status. The first and second dominant signatures are annotated at the top. STAD tumors' MSI-H and MSS status are annotated at the bottom. Hierarchical clustering was performed based on the relative contribution of signatures in each tumor. Color codes represent each

mutational signature shown. The color scale shows the contribution values of each mutational signature. For mutational signatures with known etiology, both signature and etiology are indicated. **(b)** Pair-wise statistical interaction of signatures measured by hypergeometric test: red represents co-occurrence and blue represents mutual exclusivity.

Supplementary Fig. S4. Mutational signature analysis of WES data from MSI-H and MSS COAD tumors with multivariate approach. (a) Mutational signatures of COAD tumors visualized by a heatmap divided based on distinct signature status. The first and second dominant signatures are annotated at the top. COAD tumors' MSI-H and MSS status are annotated at the bottom. Hierarchical clustering was performed based on the relative contribution of signatures in each tumor. Color codes represent each mutational signature shown. The color scale shows the contribution values of each mutational signature. For mutational signatures with known etiology, both signature and etiology are indicated. **(b)** Pair-wise statistical interaction of signatures measured by hypergeometric test: red represents co-occurrence and blue represents mutual exclusivity.

Supplementary Fig. S5. Dominant signature analysis of WGS data among STAD and COAD tumors. **(a)** The first and second dominant ID mutational signatures in STAD WGS tumors. **(b)** The first and second dominant DBS mutational signatures in STAD WGS tumors. **(c)** The first and second dominant ID mutational signatures in COAD WGS tumors. **(d)** The first and second dominant DBS mutational signatures in COAD WGS tumors**.** Dominant signatures were based on the contribution value of detected mutational signatures. For mutational signatures with known etiology, both signature and etiology are indicated.

Supplementary Fig. S6. Mutational signature analysis of STAD WGS tumors with multivariate approach. (a) Mutational signatures of STAD WGS tumors visualized by a heatmap divided based on distinct signature status. The first and second dominant signatures are annotated at the top. Hierarchical clustering was performed based on the

relative contribution of signatures in each tumor. Color codes represent each mutational signature shown. The color scale shows the contribution values of each mutational signature. For mutational signatures with known etiology, both signature and etiology are indicated. **(b)** Pair-wise statistical interaction of signatures measured by hypergeometric test: red represents co-occurrence and blue represents mutual exclusivity.

Supplementary Fig. S7. Mutational signature analysis of COAD WGS tumors with multivariate approach. (a) Mutational signatures of COAD tumors visualized by a heatmap divided based on distinct signature status. The first and second dominant signatures are annotated at the top. Hierarchical clustering was performed based on the relative contribution of signatures in each tumor. Color codes represent each mutational signature shown. The color scale shows the contribution values of each mutational signature. For mutational signatures with known etiology, both signature and etiology are indicated. **(b)** Pair-wise statistical interaction of signatures measured by hypergeometric test: red represents co-occurrence and blue represents mutual exclusivity.

Supplementary Fig. S1

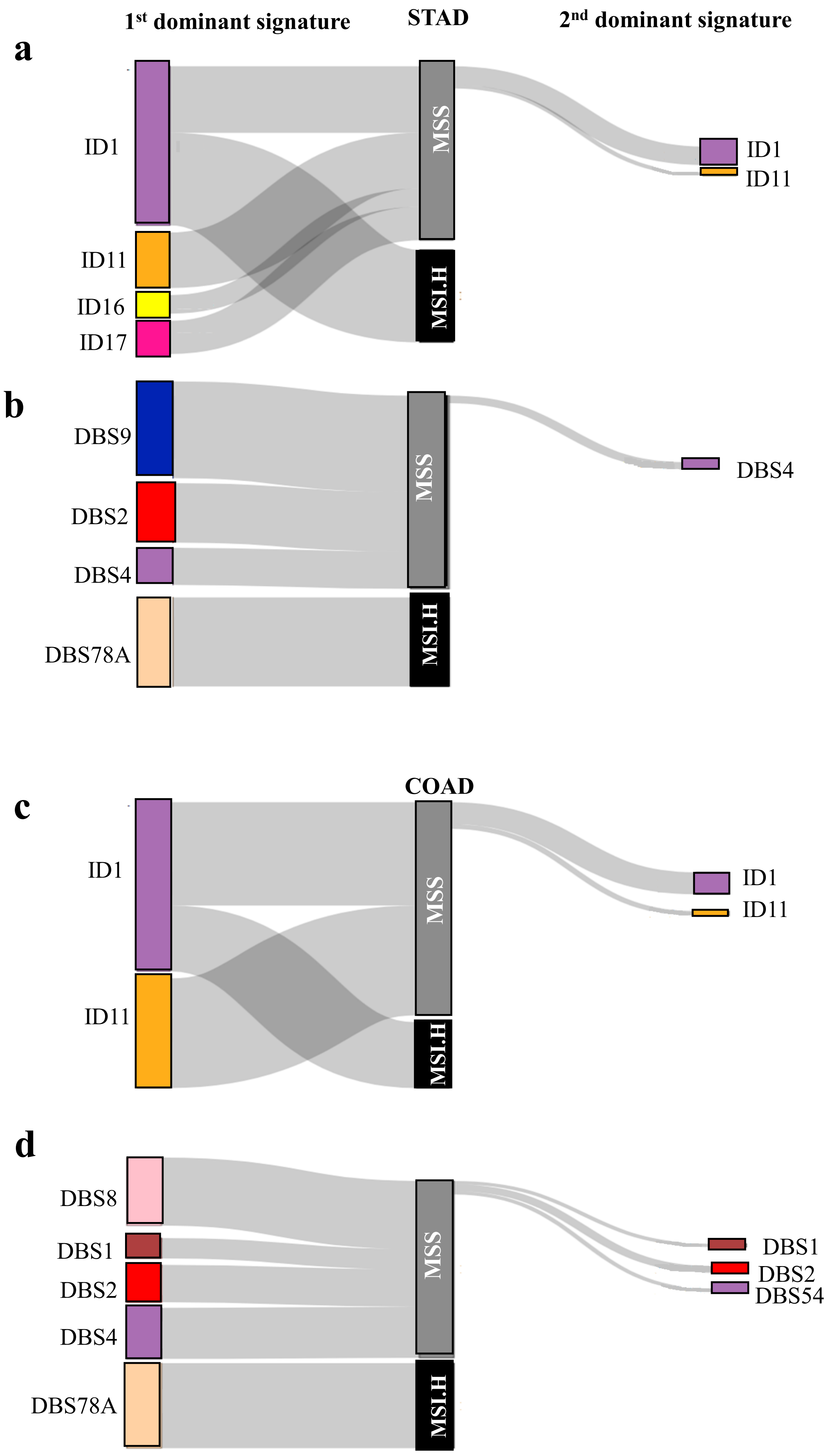

Supplementary Fig. S1

e

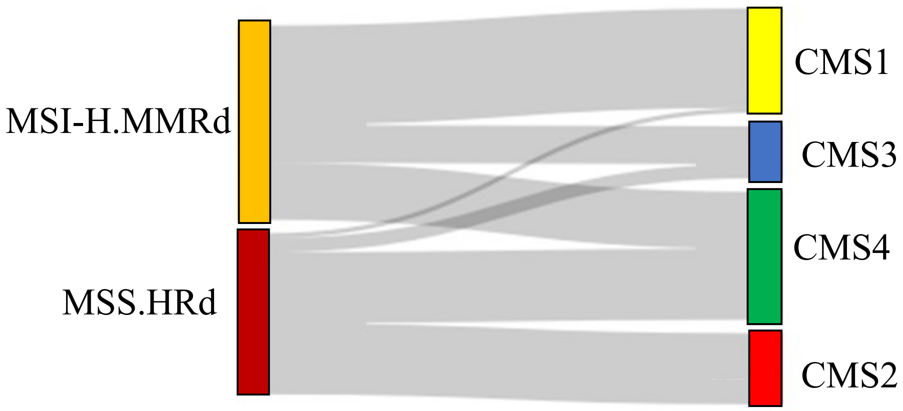

Supplementary Fig. S2

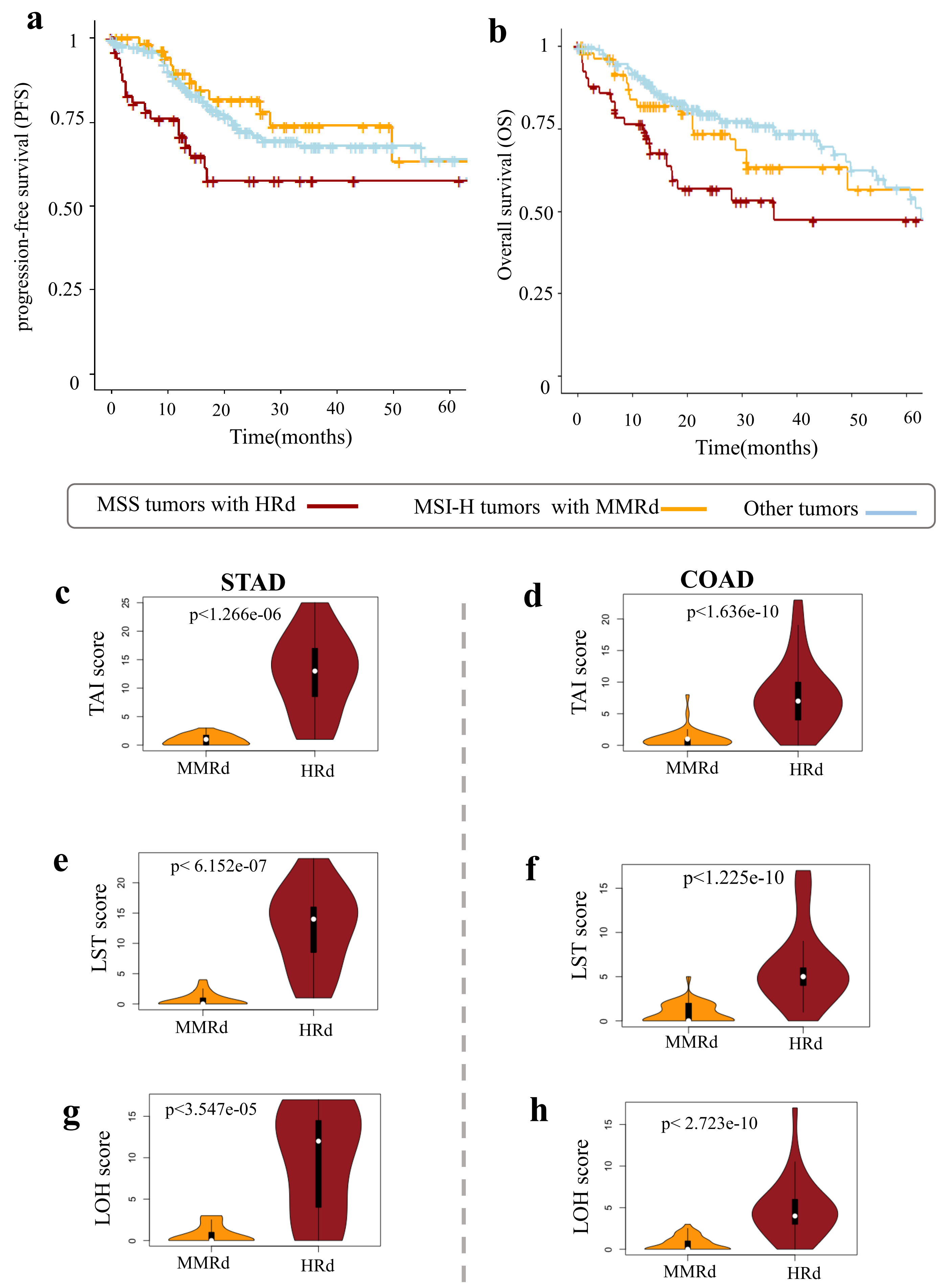

Supplementary Fig.S3

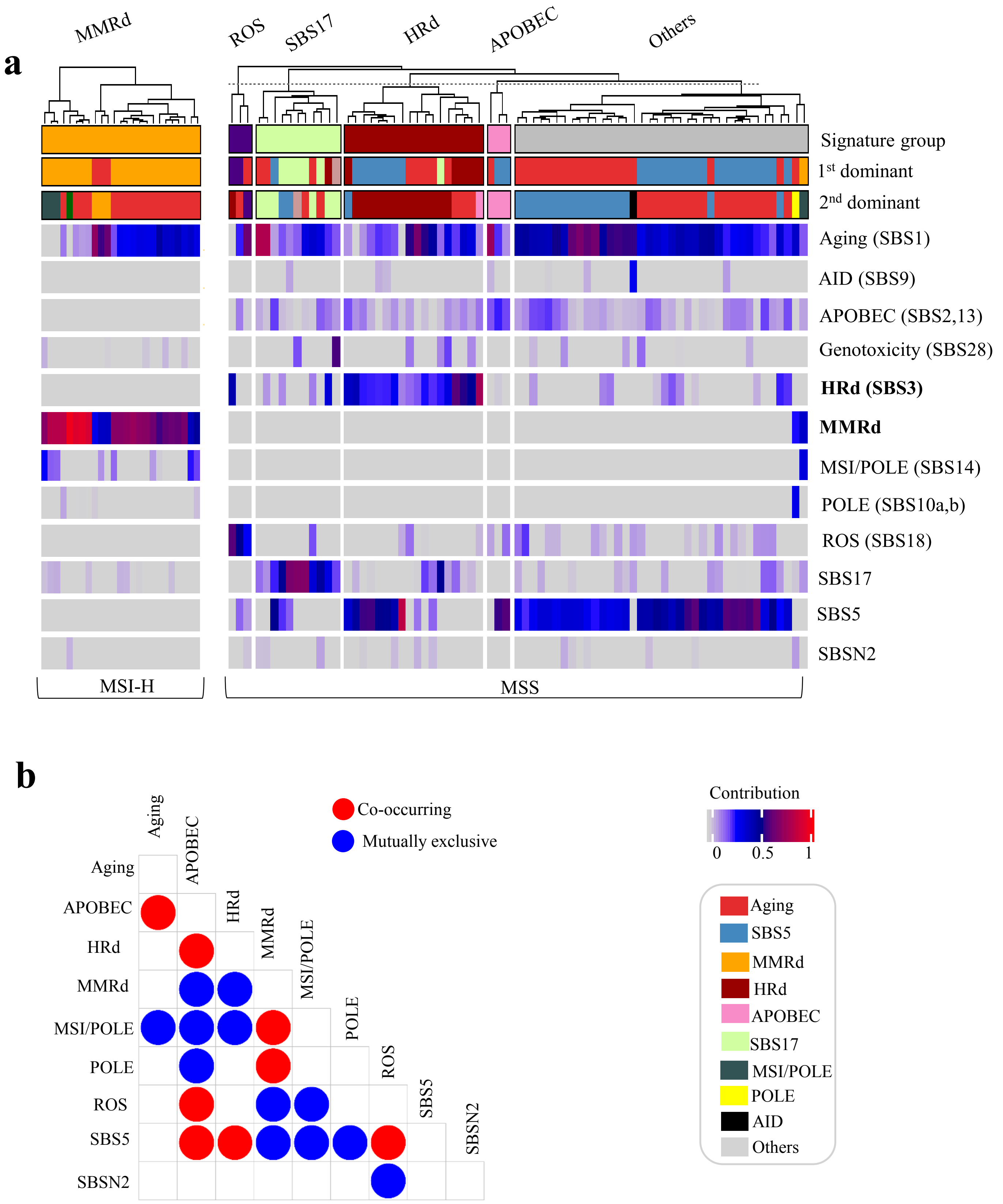

Supplementary Fig.S4

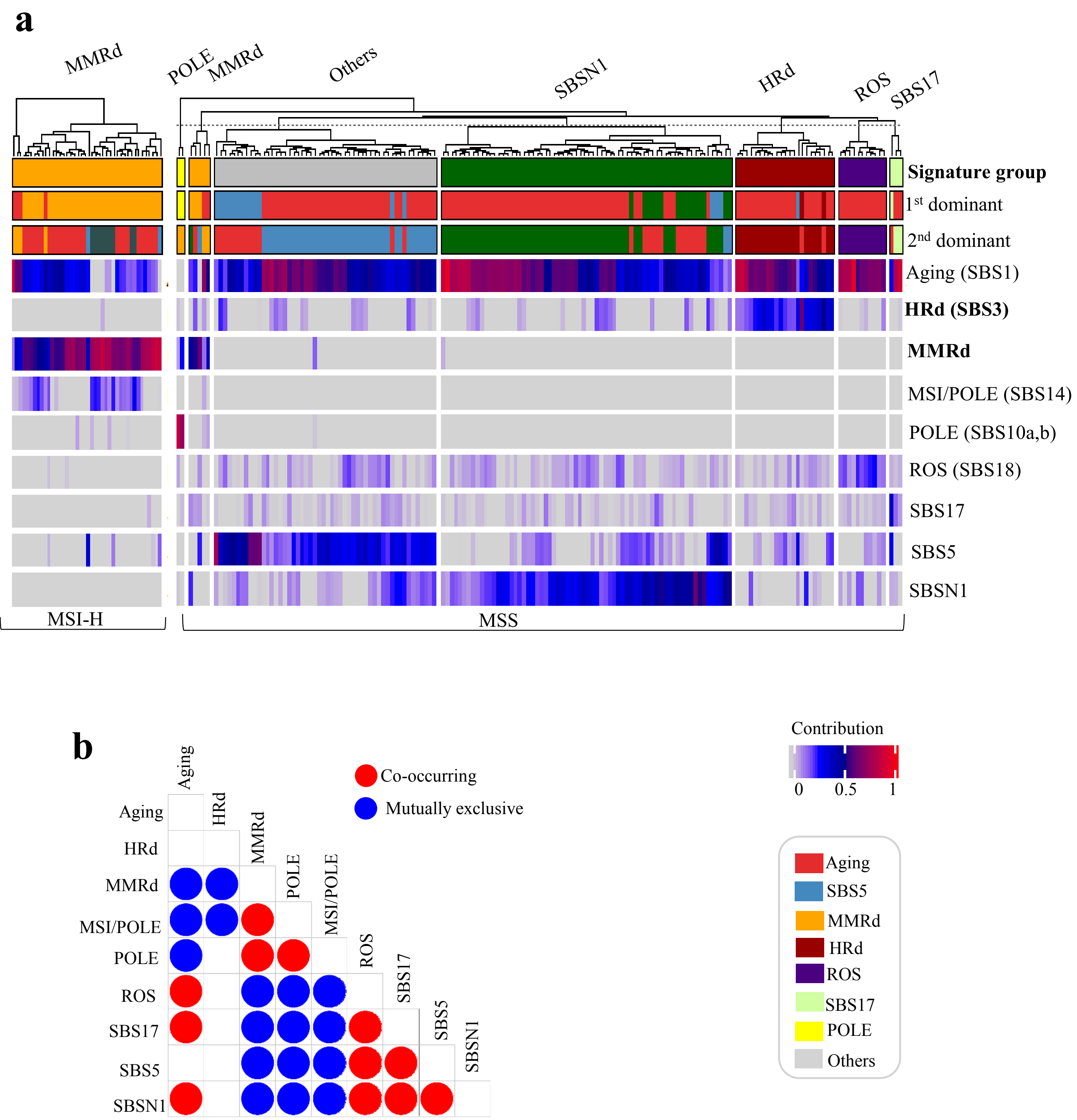

Supplementary Fig. S5

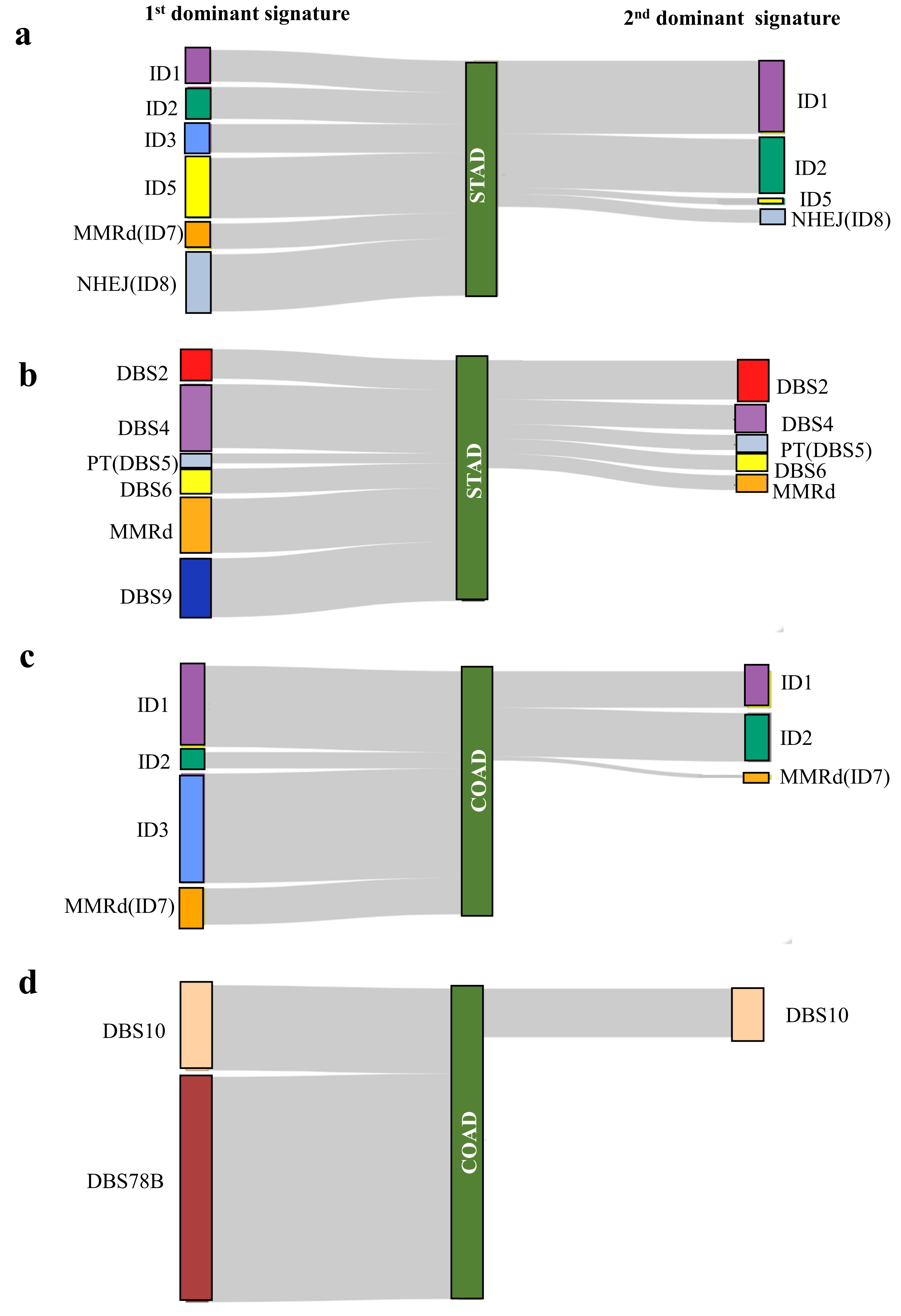

Supplementary Fig.S6

a

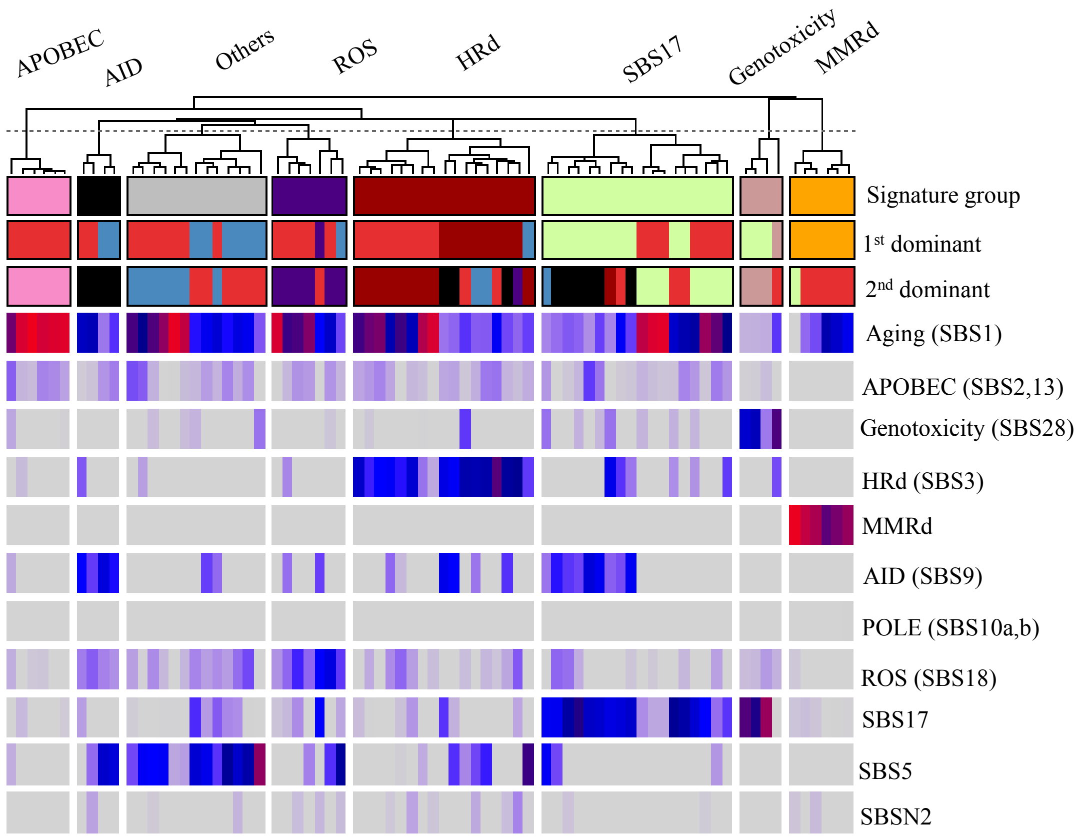

b

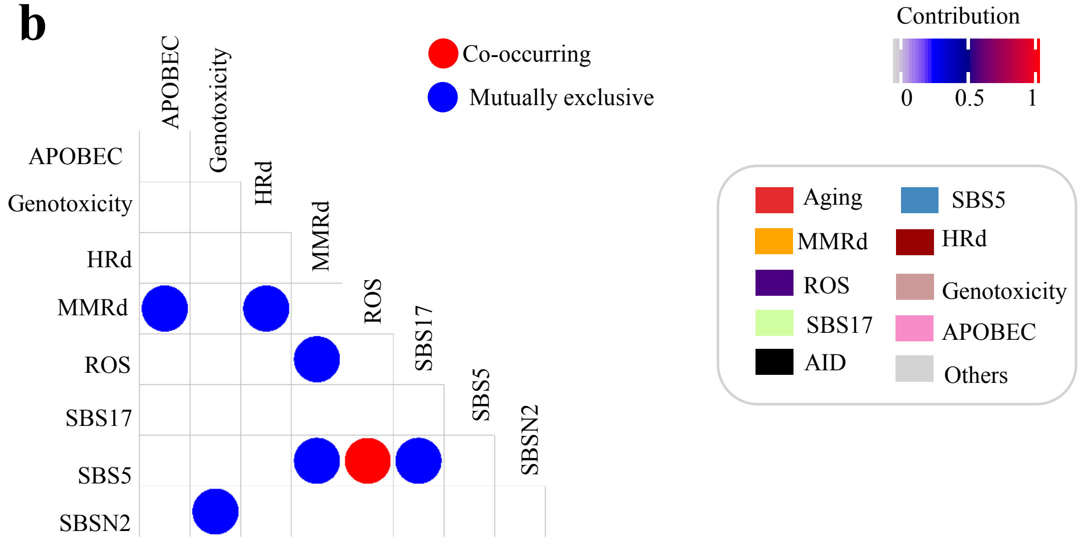

Supplementary Fig.S7

a

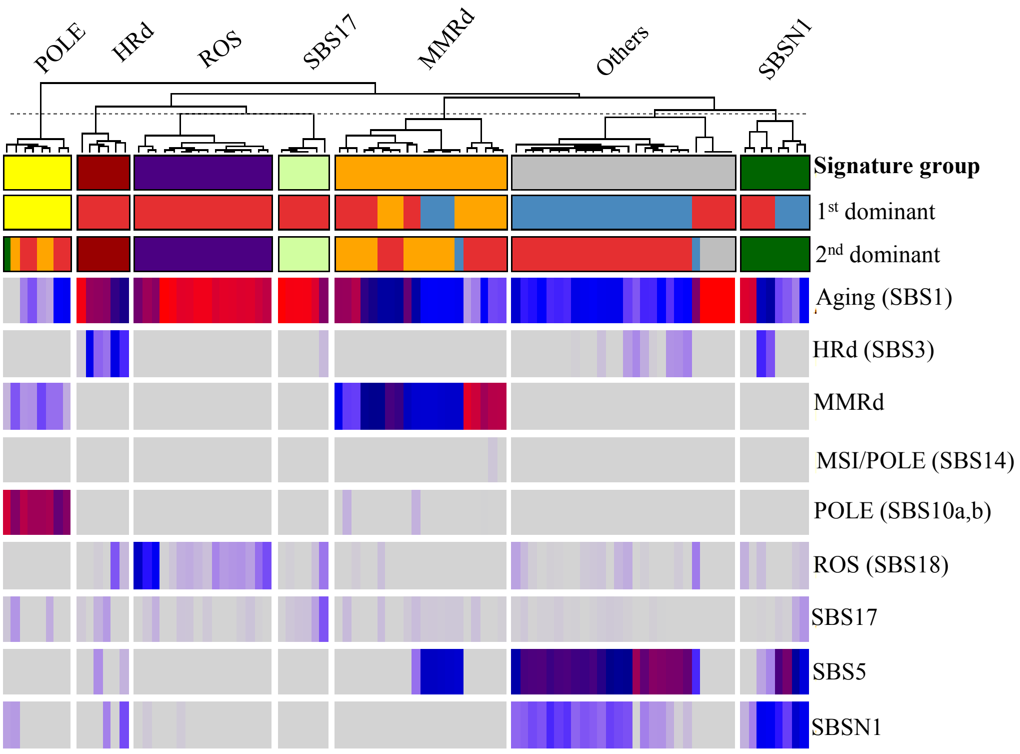

b

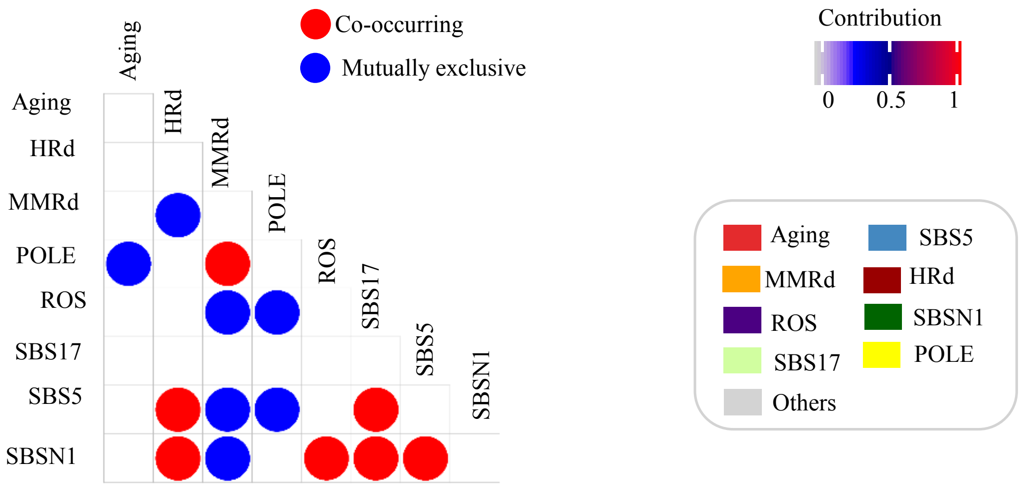

Supplement: Supplementary file 1 — Supplementary information [file 41597_2023_2331_MOESM1_ESM.pdf]
